# Exploring the Link Between Personality Traits and Self-Care Dimensions in Individuals Affected by Type 2 Diabetes Mellitus: A Comprehensive Systematic Review and Meta-Analysis

**DOI:** 10.1192/j.eurpsy.2024.635

**Published:** 2024-08-27

**Authors:** K. Dimou, E. Dragioti, G. Tsitsas, S. Mantzoukas, M. Gouva

**Affiliations:** ^1^Research Laboratory Psychology of Patients, Families and Health Professionals, Department of Nursing, School of Health Sciences, University of Ioannina, Ioannina; ^2^Department of Economy and Sustainable Development, Harokopio University, Athens; ^3^Research Laboratory Integrated Care, Health & Well-being, Department of Nursing, School of Health Sciences, University of Ioannina, Ioannina, Greece

## Abstract

**Introduction:**

Type 2 diabetes mellitus (T2DM) is a prevalent, chronic metabolic disorder that exerts diverse effects on individuals’ physical and psychological well-being.

**Objectives:**

Our aim was to investigate the potential correlation between personality traits and self-care aspects among individuals living with T2DM.

**Methods:**

We conducted a thorough search in PsycINFO, CINAHL, and PubMed/Medline for peer-reviewed articles from inception to January 9, 2023. Following PRISMA guidelines, two reviewers independently screened, extracted data, and assessed bias. We used random-effects meta-analysis for pooling estimates

**Results:**

We identified 23 studies meeting our inclusion criteria. Openness, conscientiousness, and agreeableness were linked to better foot care compliance (OR = 2.53, 95% CI = 1.49-4.28; OR = 1.84, 95% CI = 1.10-3.08; and OR = 2.07, 95% CI = 1.23-3.48, respectively). Openness was also associated with improved overall self-care behaviors (OR = 2.00, 95% CI = 1.17-3.41), while conscientiousness reduced smoking likelihood (OR = 0.96, 95% CI = 0.93-0.99), and agreeableness enhanced medication adherence (OR = 1.68, 95% CI = 1.34-2.31). However, extraversion and neuroticism were linked to lower medication adherence (OR = 0.77, 95% CI = 0.61-0.96 and OR = 0.51, 95% CI = 0.40-0.65, respectively). Neuroticism also negatively affected overall self-care behaviors (OR = 0.67, 95% CI: 0.55-0.81).

**Image:**

**Figure:**
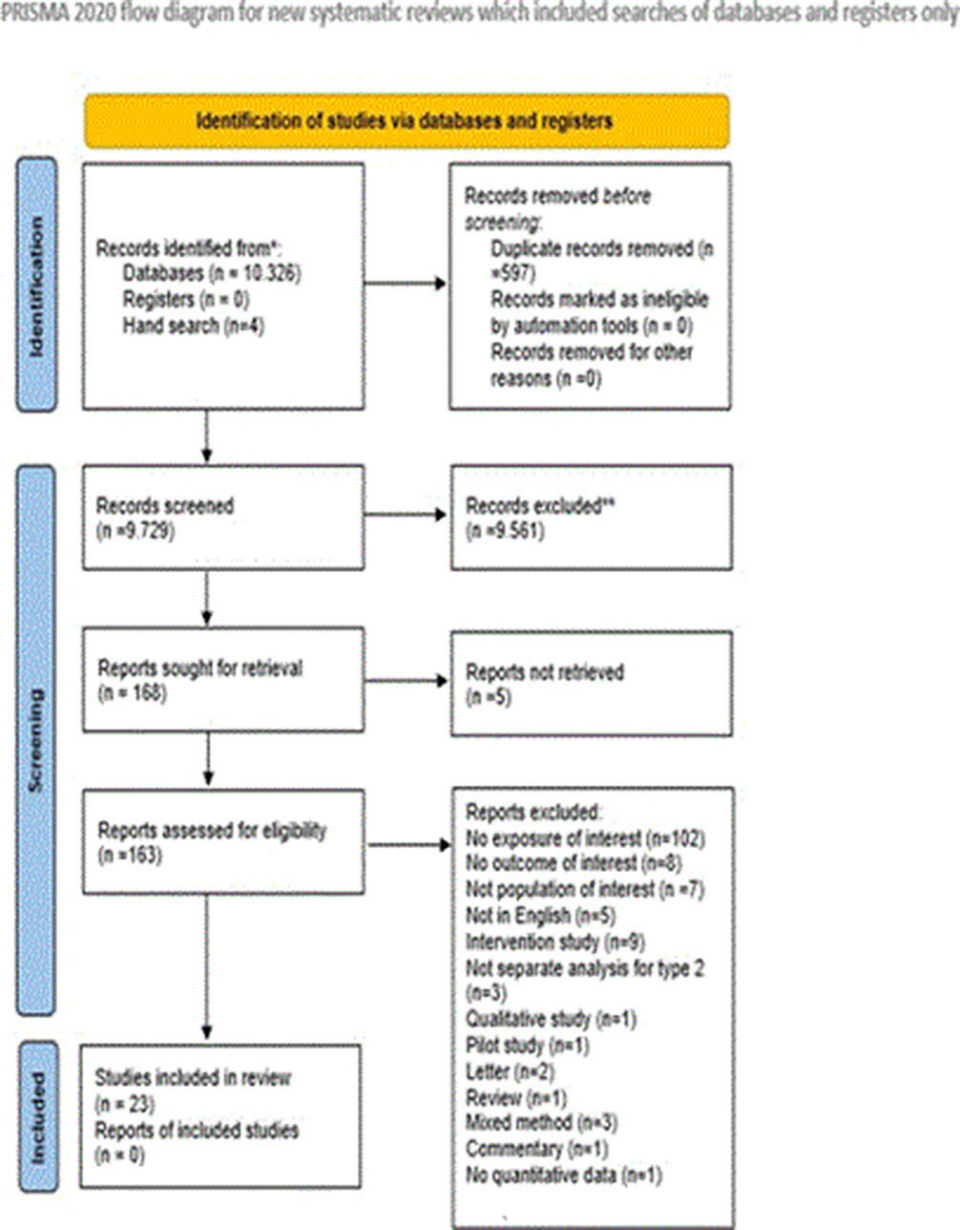

**Conclusions:**

Personality traits should be considered when addressing self-care in T2DM patients.

**Disclosure of Interest:**

None Declared

